# Supplementary material for: A conserved ankyrin repeat-containing protein regulates conoid stability, motility and cell invasion in Toxoplasma gondii
Source: Nat Commun. 2017 Dec 21;8:2236. doi: 10.1038/s41467-017-02341-2 (PMC5740107; doi:10.1038/s41467-017-02341-2)
Supplement: Supplementary file 1 — Supplementary Information [file 41467_2017_2341_MOESM1_ESM.pdf]

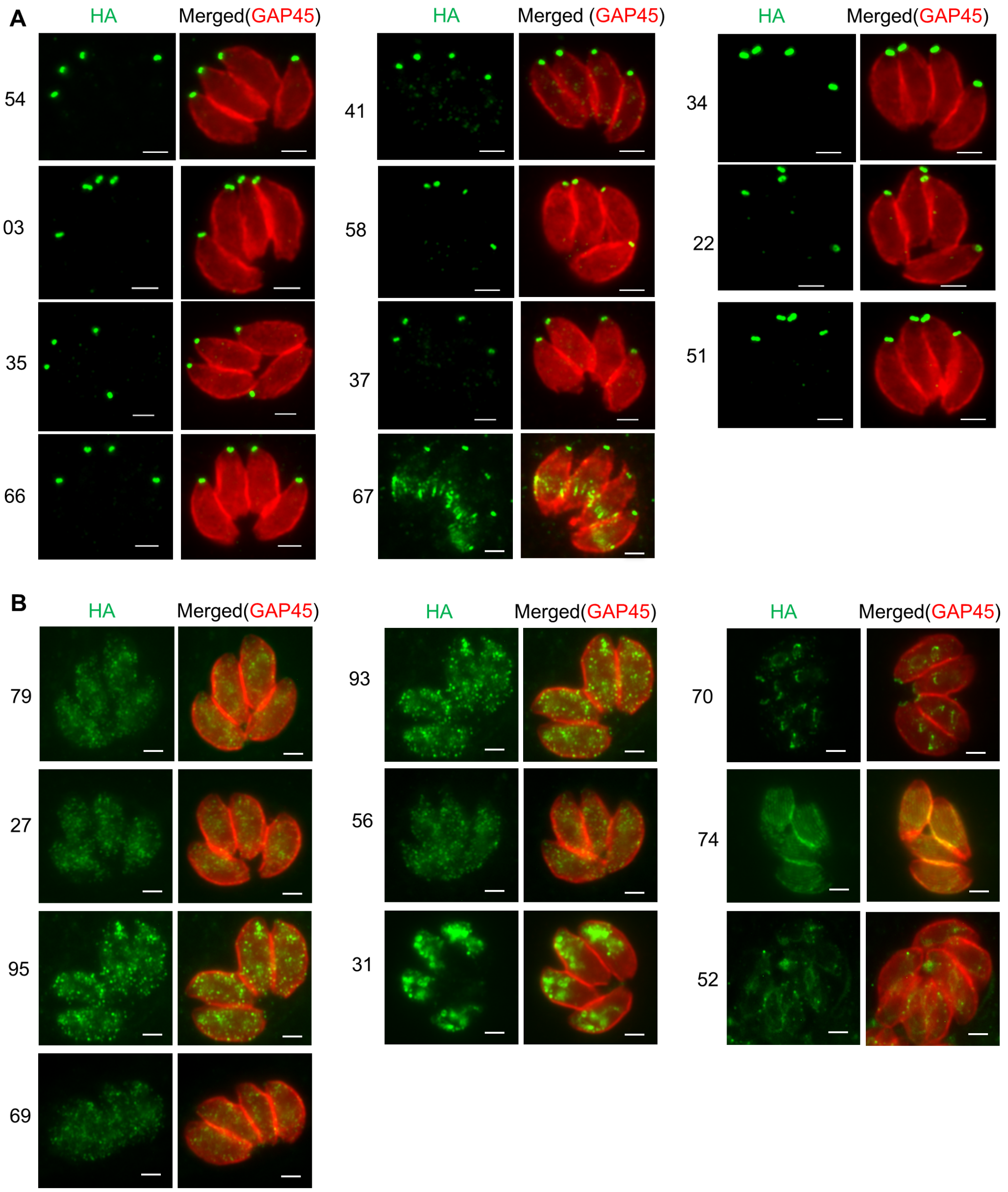

Supplementary Fig. 1. Localization of 21 hypothetical proteins by CRISPR tagging and IFA microscopy.

A. Candidate proteins localized to the apical complex.

B. Candidate proteins localized to other cellular compartments.

The genes were tagged with 6HA by CRISPR tagging technology, and IFA was performed with mouse anti-HA (green) and rabbit anti-GAP45 (red) and Alexa Fluor conjugated secondary antibodies. The candidate protein numbers (#) and other information are listed in Supplementary Table 1. Scale bar = 2µm.

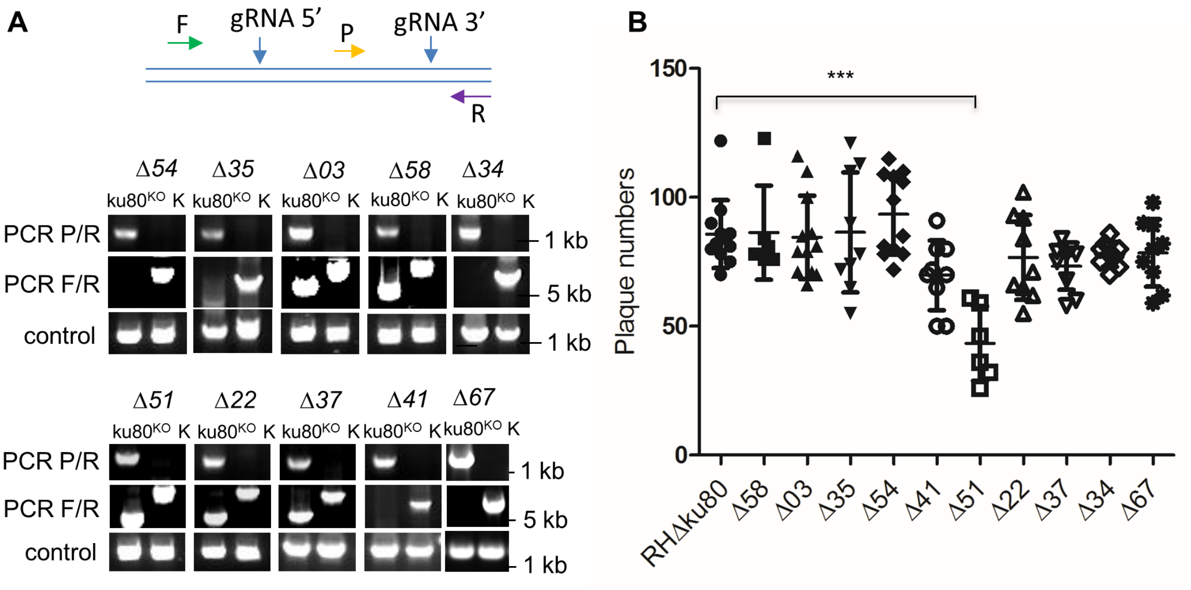

Supplementary Fig. 2. Essentiality test of the proteins localized to the apical complex in *T. gondii*.

A. A double CRISPR sgRNA strategy was used to delete the genes, and diagnostic PCR was performed as shown in the diagram. PCR with F and R primers tested the insertion of a DHFR-mCherry cassette at the targeted gene locus, and PCR with P and R primers tested the presence or absence of the endogenous gene. *CDPK1* was used as a PCR control. The knockouts (Δ) candidate numbers (see Supplementary Fig 1A and Supplementary Table 1) are listed above the PCR results, and the PCR primers for each gene are listed in Supplementary Table 4. Lane markers: $ku80^{KO}$ indicates the parental strain and K indicates the individual knockouts.

B. Plaque numbers formed by the knockouts comparing to the parental strain $ku80^{KO}$. ***, *P<0.0001* was significant only for #51 (apical polar ring protein (APR1)) vs. the parental $ku80^{KO}$ control. Kruskal Wallis comparison test with Dunn's multiple comparison test. Data shown are form three independent experiments with three replicates each (n=9).

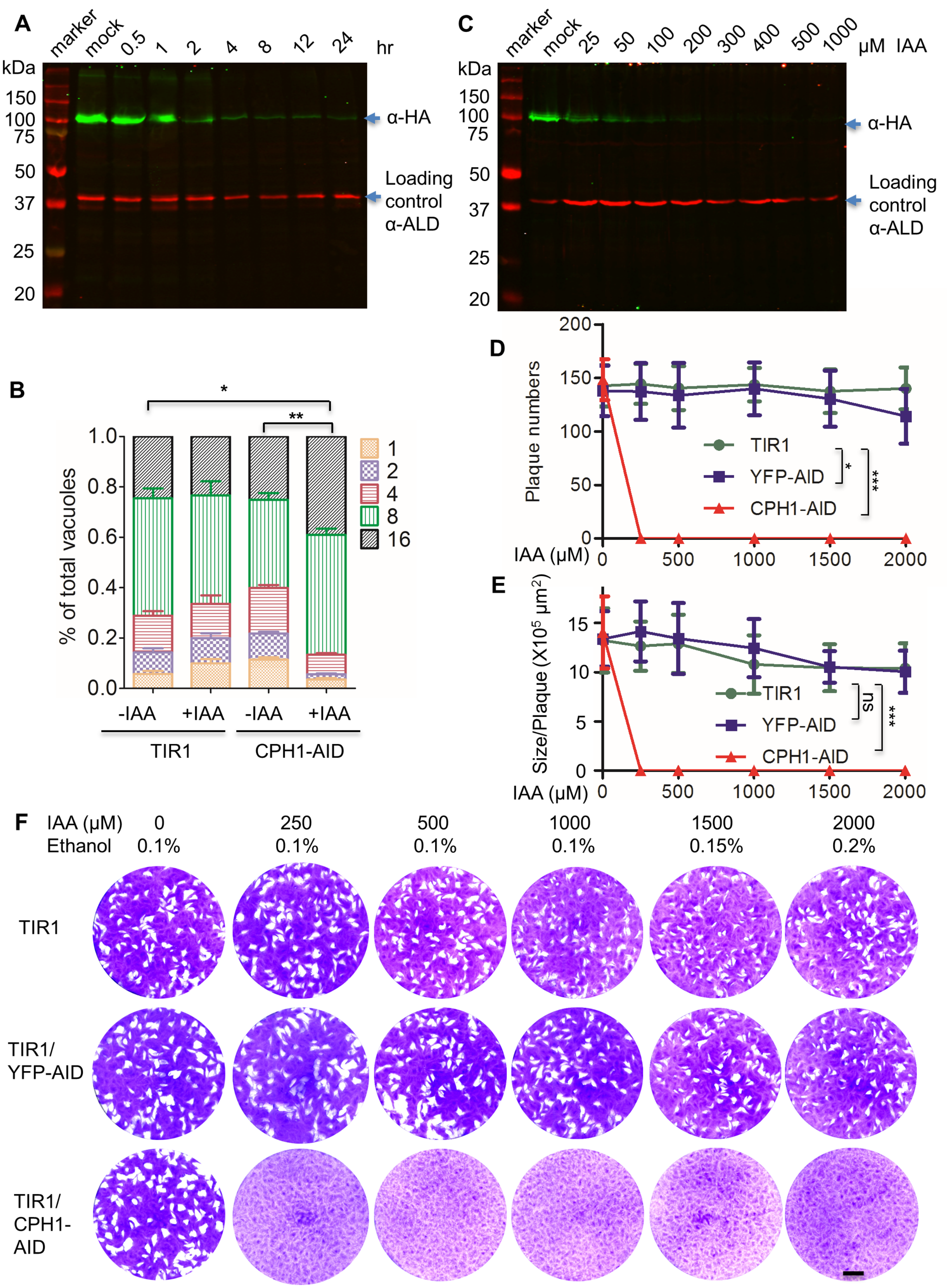

A
marker
mock
0.5
1
2
4
8
12
24
hr
kDa
150
100
75
50
37
25
20
α-HA
Loading control α-ALD
C
marker
mock
25
50
100
200
300
400
500
1000
μM IAA
kDa
150
100
75
50
37
25
20
α-HA
Loading control α-ALD
B
*
**
% of total vacuoles
1.0
0.8
0.6
0.4
0.2
0.0
1
2
4
8
16
-IAA
+IAA
-IAA
+IAA
TIR1
CPH1-AID
D
Plaque numbers
200
150
100
50
0
TIR1
YFP-AID
CPH1-AID
*
***
IAA (μM)
0
500
1000
1500
2000
E
Size/Plaque (X10^5 μm^2)
15
10
5
0
TIR1
YFP-AID
CPH1-AID
ns
***
IAA (μM)
0
500
1000
1500
2000
F
IAA (μM) 0 250 500 1000 1500 2000
Ethanol 0.1% 0.1% 0.1% 0.1% 0.15% 0.2%
TIR1
TIR1/ YFP-AID
TIR1/ CPH1- AID

Supplementary Fig. 3. Parasite replication of the TIR1 parental line and CPH1-AID line with and without auxin induction.

A. The full blot for Figure 2B with the green (α-HA) and red (α-ALD) channels combined. Degradation of CPH1-AID-3HA triggered by addition of 500 µM auxin (+IAA) vs. mock, 0.1% ethanol (-IAA) for different times (hr). Western blot was detected with mouse a-HA for CPH1-AID-3HA and rabbit anti-aldolase (a-ALD) antibodies serving as a loading control, and subsequently detected using anti-mouse and anti-rabbit secondary antibodies conjugated with LICOR C800 (green) and C680 (red) and a LICOR odyssey imaging system. A representative bot was shown from three independent experiments. Marker, precision plus protein^{TM} dual color standards (BioRad), and the ladder sizes were shown by the left side.

B. Parasites were allowed to invade HFF monolayers for 1 hr, washed to remove extracellular parasites, and allowed to grow for 24 hr during treatment with vehicle control (0.1% alcohol) or 500 µM auxin (IAA) in 0.1% alcohol. Monolayers were fixed and stained with antibodies against GAP45 and examined by epifluorescence microscopy. The number of parasites per vacuole (1, 2, 4, 8 or 16) was determined for >200 cells per group and plotted as a fraction of the total. Data shown are a composite of all three experiments with three technical replicates each (n=9). Analyzed using two-way ANOVA with Tukey's multiple comparison test. In comparing TIR1 -IAA vs. CPH1-AID +IAA (*), P = 0.0008 for 16 parasites / vacuole, and not significant for 8 parasites / vacuoles, while comparison of CPH1-AID -IAA vs CPH1-AID +IAA (**) P = 0.0013 for 16 parasites / vacuole and P =0.0042 for 8 parasites / vacuole.

C. Analysis of degradation of CPH1-AID-3HA in response to different concentrations of IAA. Samples were treated for 4 hr with different concentrations of IAA (listed at the top) each containing 0.1% ethanol. Mock was treated with 0.1% ethanol for 4 hr. Western blot was detected with mouse anti-HA epitope (α-HA) and rabbit anti-aldolase (α-ALD) primary antibodies followed by LICOR conjugated secondary antibodies. Both red and green channels were exposed on the same image. One representative blot is shown from two independent experiments. Marker, precision plus protein^{TM} dual color standards (BioRad).

D, E. Parasite growth under different concentration of IAA as shown by plaquing. Parasite lines were inoculated (250 parasites in 4 ml D10 media) into 6-well plates containing HFF monolayers and grown for 7 days with different concentrations of IAA (as indicated). After 7 days of growth, monolayers were fixed with 75% ethanol and stained by 0.1% crystal violet. Two independent experiments with triplicates for each cell line were performed (N = 6). **D**. Plaque numbers for each treatment were plotted as Mean ± S.D. and analyzed using two-way ANOVA with Dunnett's multiple comparison. In comparing control TIR1 (+IAA) line vs. YFP-AID line (+IAA), there was a small but significant decrease in the number of plaques for YFP-AID only at the highest concentration of auxin (i.e. 2,000 µM) P = 0.0396 (*). In contrast, when comparing the control TIR1 (+IAA) line vs. CPH1-AID (+IAA), there was an absence of plaques formed by the CPH1-AID line at all concentrations of IAA, P < 0.0001 (***). Data are combined from two independent experiments, triplicate wells each (n=6). **E**. Plaque sizes for each parasite line were analyzed using Zeiss Axio Observer D.1 microscope and Zeiss software Zen 2.3 pro. Mean ± S.D. were plotted (two independent experiments, triplicate

wells, and six plaques analyzed for each well (N = 36)), and analyzed using two-way ANOVA with Dunnett's multiple comparison. In comparing the control TIR1 (+IAA) line vs. YFP-AID line (+IAA), there was no significant difference in plaque size for all the points. In contrast, when comparing the control TIR1 (+IAA) line vs. CPH1-AID (+IAA), due to the absence of plaques for CPH1-AID (plaque size = 0) the size differences were significant for all concentrations of IAA, $P < 0.0001$ (***).

F. Representative images from **D, E** are shown for each parasite line. Concentration of IAA (μM) and ethanol (%) are indicated above the images. Scale = 0.5 cm.

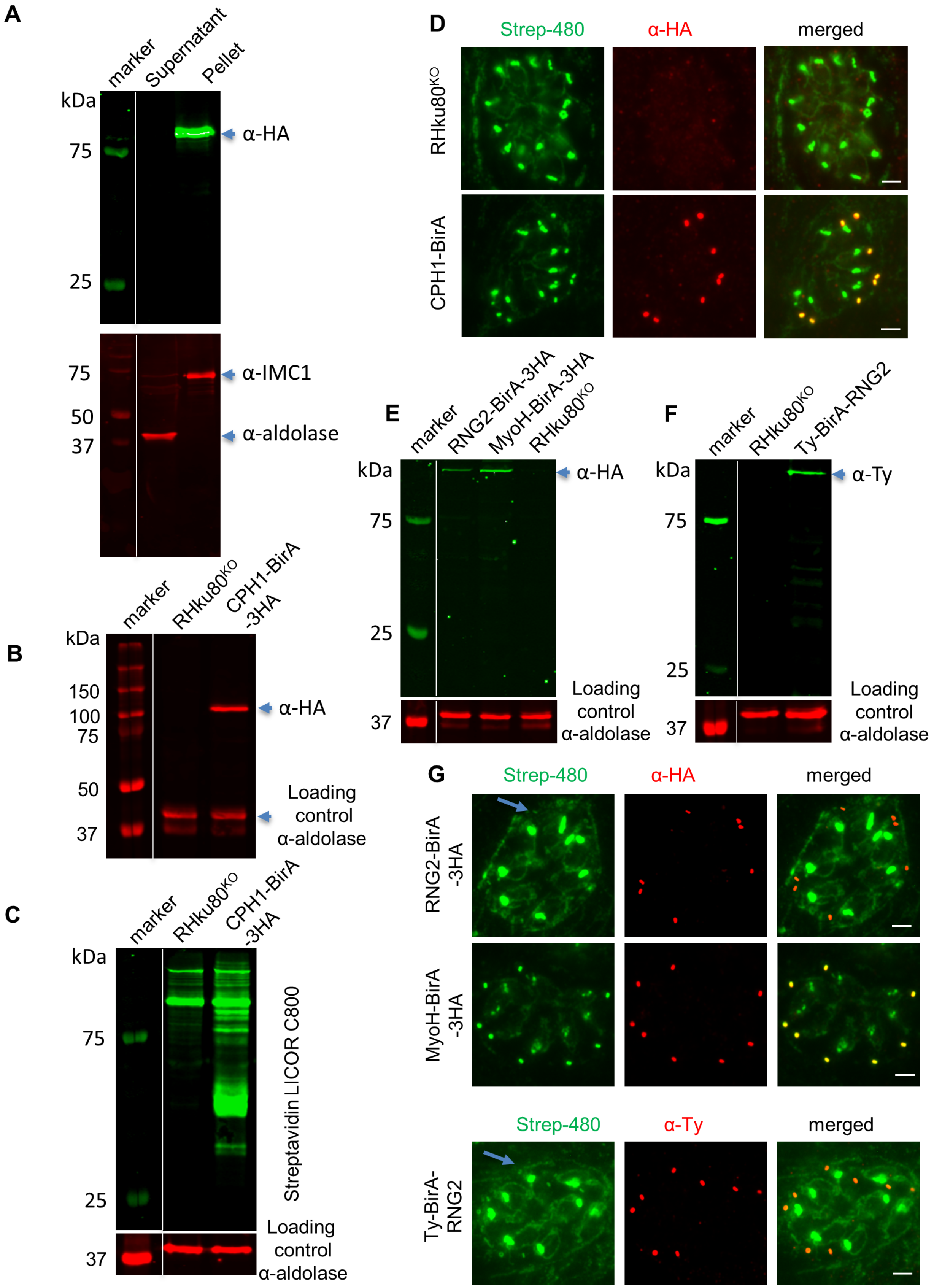
A
marker
Supernatant
Pellet
kDa
75
25
α-HA
75
50
37
α-IMC1
α-aldolase
B
marker
RHku80KO
CPH1-BirA -3HA
kDa
150
100
75
50
37
α-HA
Loading control α-aldolase
C
marker
RHku80KO
CPH1-BirA -3HA
kDa
75
25
37
Streptavidin LICOR C800
Loading control α-aldolase
D
Strep-480
α-HA
merged
RHku80KO
CPH1-BirA
E
marker
RNG2-BirA-3HA
MyoH-BirA-3HA
RHku80KO
kDa
75
25
37
α-HA
Loading control α-aldolase
F
marker
RHku80KO
Ty-BirA-RNG2
kDa
75
25
37
α-Ty
Loading control α-aldolase
G
Strep-480
α-HA
merged
RNG2-BirA -3HA
MyoH-BirA -3HA
Strep-480
α-Ty
merged
Ty-BirA-RNG2

Supplementary Fig. 4. Generation and verification of BirA fusion lines.

A. CPH1 was insoluble in detergent lysates of *T. gondii*. Western blot using mouse α-HA for detection of CPH1-AID-3HA, rabbit α-aldolase serving as a control for soluble proteins in the supernatant and mouse α-IMC1 serving as a control for insoluble proteins in the pellet, and visualized with LICOR secondary antibody reagents. Samples were lysed in 1% Triton X-100 in PBS for 10 min on ice, then centrifuged at 20,000*g* for 10 min at 4°C.

B. Confirmation of CPH1-BirA line by Western blot. CPH1 was tagged with BirA-3HA at the C-terminus using CRISPR tagging technology, and parasite lysates were resolved by SDS-PAGE and detected with mouse α-HA and rabbit α-aldolase (loading control) visualized with LICOR secondary antibody reagents.

C. Detection of biotinylated proteins by Western blot. RHku80^{KO} and CPH1-BirA lines were grown in 180 µM D-biotin for 20 hr, and lysates were Western blotted using streptavidin LICOR C800. Aldolase served as a loading control and was detected by Western blotting as in B.

D. Targets that were biotinylated by CPH1-BirA localized to the conoid. Intracellular parasites grown in HFF cells and treated with 180 µM D-biotin for 20 hr were fixed and stained with streptavidin Alexa Fluor-488 (green) and rabbit anti-HA visualized with anti-rabbit Alexa Fluor -594 (red). Scale bar = 2 µm

E and F. Generation and verification of RNG1-BirA, BirA-RNG2, and MyoH-BirA by Western blot. Mouse α-HA was used to detect RNG2-BirA and MyoH-BirA lines, and mouse α-Ty was used to detect BirA-RNG2. Rabbit α-aldolase served as a loading control and was detected by Western blotting as in B.

G. Targets that were biotinylated by RNG2-BirA, BirA-RNG2, and MyoH-BirA localized to the conoid. The assay was performed as that for D. The blue arrows indicate the position of the conoid. Scale bar = 2 µm.

Markers appendix to the full blots in A, B, C, E and F, precision plus protein^{TM} dual color standards (BioRad).

A

GO Terms – Cellular Compartment

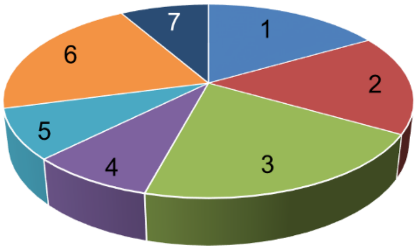

1	IMC
2	Pellicle
3	Protein complex
4	Myosin complex
5	Actin cytoskeleton
6	Macromolecular complex
7	Cytoskeleton part

B

GO Terms – Molecular Function

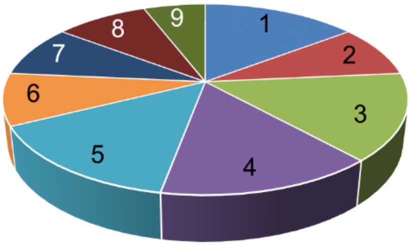

1	Nucleoside-triphosphatase activity
2	GTPase activity
3	Pyrophosphatase activity
4	Hydrolase activity, for phosphorus-containing anhydrides
5	Hydrolase activity, acting on acid anhydrides
6	GTP binding
7	Guanyl ribonucleotide binding
8	Guanyl nucleotide binding
9	Motor activity

Supplementary Fig. 5. Gene ontology (GO) analyses for annotated proteins in the CPH1-RNG2-MyoH interactome.
A. Classification of Supplementary Data 3 based on Cellular Compartment.
B. Classification of Supplementary Data 3 based on Molecular Function.
The numbers listed in the table refer to the numbers used in the pie chart. Gene ontology (GO) Terms were based on TOXODB (http://toxodb.org). GO Terms based on cellular compartment with *P* values < 0.004 and with Bonferroni corrections < 0.06 were used for plotting a pie chart, and the GO terms on molecular function with *P* values < 0.004 and with Bonferroni corrections < 0.1 were used for plotting a pie chart.

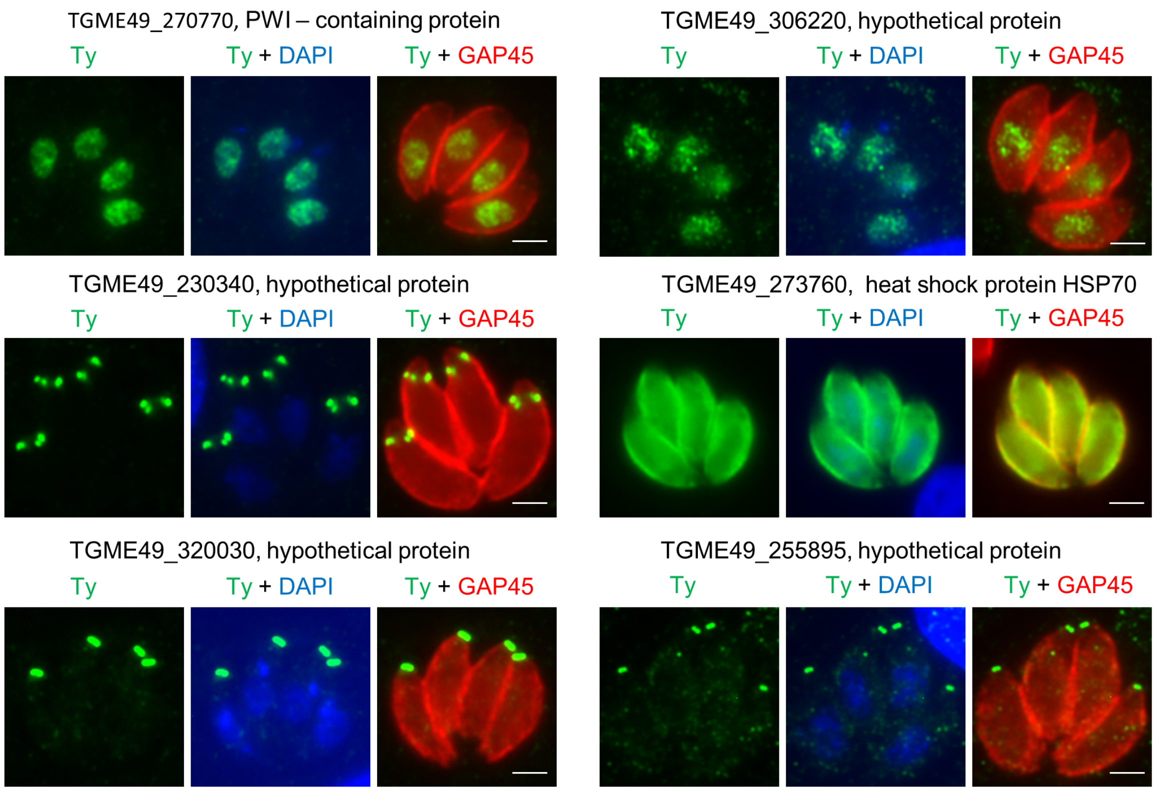

Supplementary Fig. 6. Localization of proteins identified in the CPH1-RNG2-MyoH interactome. Proteins were tagged with 2Ty using a CRISPR tagging technology and expressed in the CPH1-AID line. Proteins were visualized by IFA using antibodies against Ty (green) and GAP45 (red). DAPI was used to stain the nucleus. Scale = 2 μm.

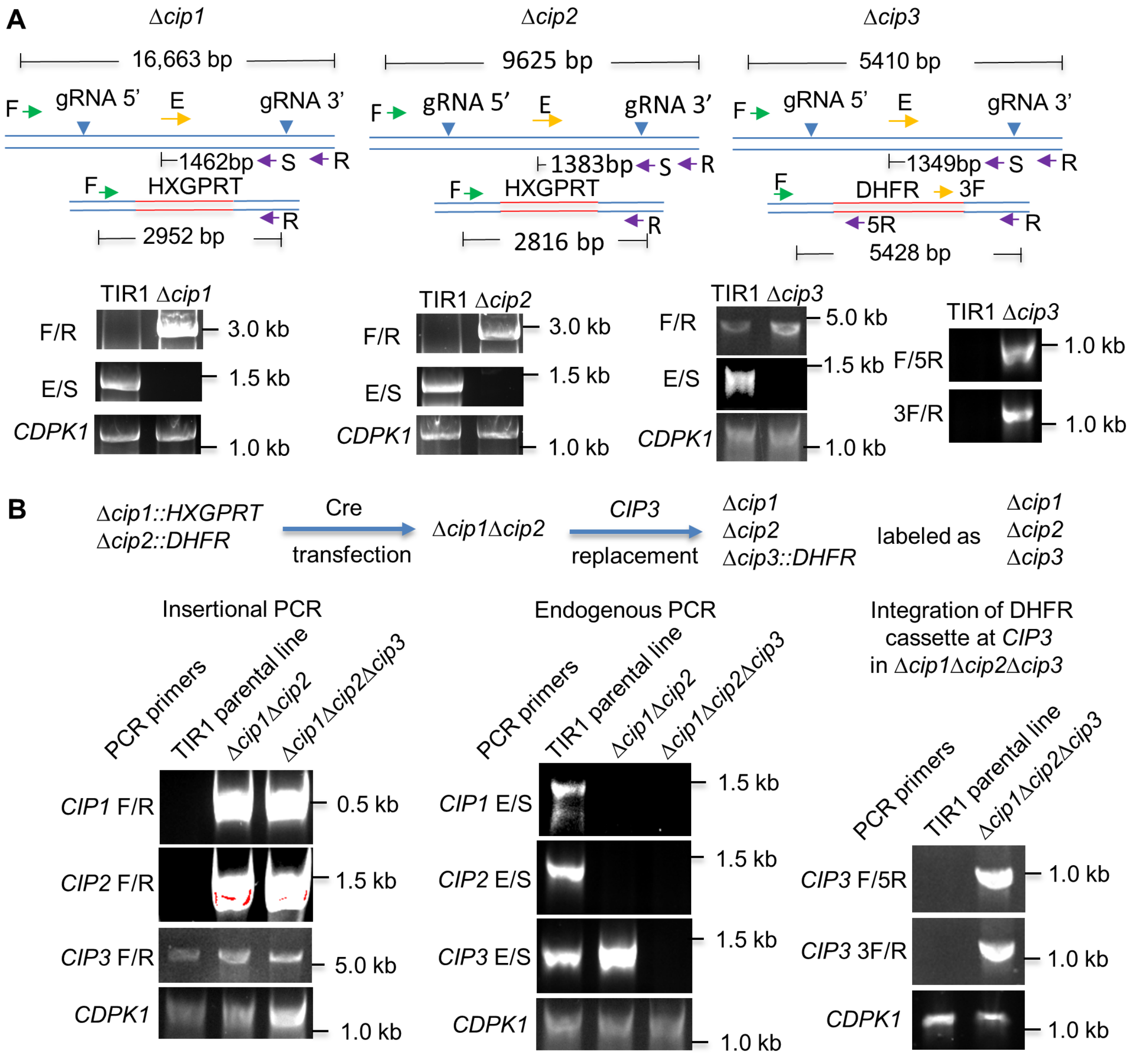

Supplementary Fig. 7. Generation and verification of *CIP1*, *CIP2* and *CIP3 knockouts*.

A. Strategies of knockout generation and diagnostic PCR are illustrated at the top. For each gene, double sgRNAs were designed for CRISPR targeting and these are denoted as gRNA5' and gRNA3'. PCR testing with primers F/R confirmed the insertion of resistance cassette (insertional PCR), and PCR testing with primers E/S confirmed the presence or absence of the endogenous gene (endogenous PCR). Integration of the DHFR cassette was confirmed using primers *CIP3* F/5R and *CIP3* 3F/R (integration PCR). The sizes of PCR products are shown below the diagrams. TIR1 refers to the parental line.

B. Generation and verification of double and triple knockouts. The workflow for generation of the lines is shown in the upper part, and similar strategies to confirm gene knockouts were performed as described for the single knockouts in **A.**

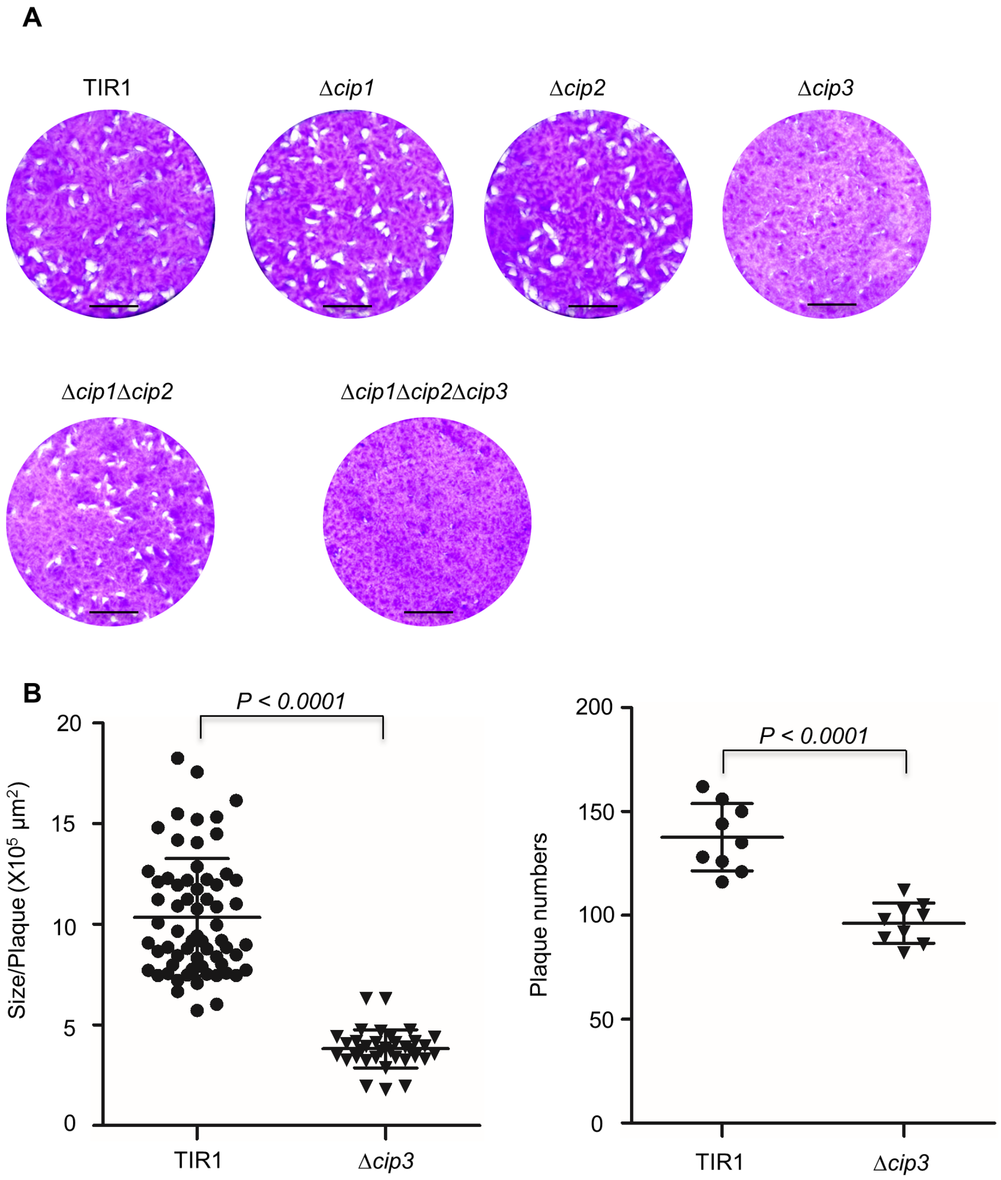

Supplementary Fig. 8 Growth phenotype of single, double and triple knockouts in CIP proteins.

A. Parasites were grown in HFF monolayers for 7 days, fixed, and stained with crystal violet. Images are shown for one example for each line. The experiment was repeated three times with similar results. There was no obvious plaque defect for Δ*cip1* or Δ*cip2*.
B. Plaque size and numbers were measured for the single knockout of Δ*cip3*. Mean ± SEM (3 experiments, each with 3 technical replicates, n=9). Scale = 0.5 cm. *P < 0.0001*, Mann-Whitney test. Quantification of plaque size and number for the double and triple mutants are show in Figure 7C.

Supplementary Table 1. Hypothetical proteins identified by the analysis of expression profiling.

Acc. No.[1]	#[2]	Localization (Tg)	Name	Tg[3]	Nc	Et	Pf	Cp	Bb	Tp	Gn
254870	54	apical complex		+	+	+	-	-	-	-	-
222350	35	apical complex		+	+	+	+	+	+	+	+
320030	03	apical complex		+	+	+	+	-	-	-	-
258090	58	apical complex		+	+	+	-	-	-	-	-
223790	79	punctate		+	+	+	+	+	+	+	-
227000	27	cytosol		+	+	+	+	-	-	-	-
269330	69	cytosol		+	+	+	+	+	+	+	+
295420	95	punctate		+	+	+	-	-	-	-	-
315510	51	apical complex	APR1[5]	+	+	+	-	-	-	-	-
246720	67	partially apical		+	+	+	+	+	-	-	-
293540	93	punctate		+	+	+	-	+	-	-	-
313780	37	apical complex		+	+	-	-	-	-	-	-
266630	66	apical complex	CPH1[4]	+	+	+	+	+	+	+	+
245640	56	punctate		+	+	+	-	-	-	-	-
312150	31	punctate		+	+	+	-	-	-	-	-
226990	22	apical complex		+	+	-	-	-	-	-	-
234270	34	apical complex		+	+	-	-	-	-	-	-
305270	52	punctate		+	+	-	-	-	-	-	-
231070	70	basal complex		+	+	+	-	-	-	-	-
274120	41	apical complex		+	+	+	+	+	-	-	-
24874	74	cytoskeleton		+	+	-	-	-	-	-	-

0											

[1] refers to gene accession numbers for TgME49: http://ToxoDB.org

[2] refers to numbers used for the genes studied here.

[3] abbreviation for apicomplexan species, Tg, *Toxoplasma gondii*; Nc, *Neospora canicun*; Et, *Emeria tenella*; Pf, *Plasmodium falciparum*; Cp, *Cryptosporidium parvum*; Bb, *Babesia bovis*; Tp, *Theileria parva*; Gn, *Gregarina niphandrodes*. Orthologs of the hypothetical proteins were identified by searching the proteins in the HMMER website (http://www.ebi.ac.uk/Tools/hmmer/) against reference proteomes, and hits with E-values < 1e-7 were considered as orthologs. + indicates presence of the orthologs in the species, - indicates absence of the orthologs in the species.

[4] Orthologs of CPH1 in apicomplexan species were used for the phylogeny study, as listed below: *Hammondia hammondi. H. hammondi,* XP_008888425.1*; Neospora caninum, N.caninum,* XP_003880899.1; *Eimeria tenella, E.tenella,* XP_013228471.1*; Cryptosporidium parvum, C.parvum,* XP_627965.1*; Plasmodium falciparum, P.falciparum,* XP_001349132.1*; Plasmodium berghei, P.berghei,* XP_677221.1*; Gregarina niphandrodes, G.niphandrodes,* XP_011130926.1*; Theileria parva, T.parva,* XP_765776.1*; Babesia bovis, B.bovis,* XP_001610424.1.

[5] Apical polar ring protein

Supplementary Table 2. Lines used in the study.

No.	Name[1]	Genotype[2]	Aim
1	54-6HA	RHΔ*ku80*Δ*hxgprt*; *54-6HA*, *DHFR-TS:HXGPRT*	IFA
2	35-6HA	RHΔ*ku80*Δ*hxgprt*; *35-6HA*, *DHFR-TS:HXGPRT*	IFA
3	03-6HA	RHΔ*ku80*Δ*hxgprt*; *03-6HA*, *DHFR-TS:HXGPRT*	IFA
4	58-6HA	RHΔ*ku80*Δ*hxgprt*; *58-6HA*, *DHFR-TS:HXGPRT*	IFA
5	79-6HA	RHΔ*ku80*Δ*hxgprt*; *79-6HA*, *DHFR-TS:HXGPRT*	IFA
6	66-6HA	RHΔ*ku80*Δ*hxgprt*; *66-6HA*, *DHFR-TS:HXGPRT*	IFA
7	27-6HA	RHΔ*ku80*Δ*hxgprt*; *27-6HA*, *DHFR-TS:HXGPRT*	IFA
8	69-6HA	RHΔ*ku80*Δ*hxgprt*; *69-6HA*, *DHFR-TS:HXGPRT*	IFA
9	95-6HA	RHΔ*ku80*Δ*hxgprt*; *95-6HA*, *DHFR-TS:HXGPRT*	IFA
10	51-6HA	RHΔ*ku80*Δ*hxgprt*; *51-6HA*, *DHFR-TS:HXGPRT*	IFA
11	67-6HA	RHΔ*ku80*Δ*hxgprt*; *67-6HA*, *DHFR-TS:HXGPRT*	IFA
12	93-6HA	RHΔ*ku80*Δ*hxgprt*; *93-6HA*, *DHFR-TS:HXGPRT*	IFA
13	37-6HA	RHΔ*ku80*Δ*hxgprt*; *37-6HA*, *DHFR-TS:HXGPRT*	IFA
14	56-6HA	RHΔ*ku80*Δ*hxgprt*; *56-6HA*, *DHFR-TS:HXGPRT*	IFA
15	31-6HA	RHΔ*ku80*Δ*hxgprt*; *31-6HA*, *DHFR-TS:HXGPRT*	IFA
16	22-6HA	RHΔ*ku80*Δ*hxgprt*; *22-6HA*, *DHFR-TS:HXGPRT*	IFA
17	34-6HA	RHΔ*ku80*Δ*hxgprt*; *34-6HA*, *DHFR-TS:HXGPRT*	IFA
18	52-6HA	RHΔ*ku80*Δ*hxgprt*; *52-6HA*, *DHFR-TS:HXGPRT*	IFA
19	70-6HA	RHΔ*ku80*Δ*hxgprt*; *70-6HA*, *DHFR-TS:HXGPRT*	IFA
20	41-6HA	RHΔ*ku80*Δ*hxgprt*; *41-6HA*, *DHFR-TS:HXGPRT*	IFA
21	74-6HA	RHΔ*ku80*Δ*hxgprt*; *74-6HA*, *DHFR-TS:HXGPRT*	IFA
22	*Δ54*	RHΔ*ku80*Δ*hxgprt*; *Δ54:: DHFR-TS:DHFR-*	Essentiality test

		mCherry	
23	*Δ35*	RHΔ*ku80*Δ*hxgprt*; *Δ35::DHFR-TS:DHFR-mCherry*	Essentiality test
24	*Δ03*	RHΔ*ku80*Δ*hxgprt*; *Δ03::DHFR-TS:DHFR-mCherry*	Essentiality test
25	*Δ58*	RHΔ*ku80*Δ*hxgprt*; *Δ58::DHFR-TS:DHFR-mCherry*	Essentiality test
26	*Δ34*	RHΔ*ku80*Δ*hxgprt*; *Δ34::DHFR-TS:DHFR-mCherry*	Essentiality test
27	*Δ51*	RHΔ*ku80*Δ*hxgprt*; *Δ51::DHFR-TS:DHFR-mCherry*	Essentiality test
28	*Δ22*	RHΔ*ku80*Δ*hxgprt*; *Δ22::DHFR-TS:DHFR-mCherry*	Essentiality test
29	*Δ37*	RHΔ*ku80*Δ*hxgprt*; *Δ37::DHFR-TS:DHFR-mCherry*	Essentiality test
30	*Δ41*	RHΔ*ku80*Δ*hxgprt*; *Δ41::DHFR-TS:DHFR-mCherry*	Essentiality test
31	*Δ67*	RHΔ*ku80*Δ*hxgprt*; *Δ67::DHFR-TS:DHFR-mCherry*	Essentiality test
32	CPH1-AID	RHΔ*ku80*Δ*hxgprt*; *TUB1:OsTIR1-3FLAG, SAG1:CAT; CPH1-AID-3HA*	Conditional knockdown
33	CPH1-AID/MIC2-GLuc	RHΔ*ku80*Δ*hxgprt*; *TUB1:OsTIR1-3FLAG, SAG1:CAT; CPH1-AID-3HA*; *TUB1:MIC2-GLuc-c-myc, DHFR-TS:HXGPRT*	Microneme secretion
34	TIR1/MIC2-GLuc	RHΔ*ku80*Δ*hxgprt; TUB1:OsTIR1-3FLAG, SAG1:CAT; TUB1:MIC2-GLuc-c-myc, DHFR-TS:HXGPRT*	Microneme secretion
35	CPH1-AID/DsRed	RHΔ*ku80*Δ*hxgprt; TUB1:OsTIR1-3FLAG, SAG1:CAT; CPH1-AID-3HA; TUB1:SAG1-DsRed, DHFR-TS:DHFR*	Microneme secretion
36	CPH1-AID/CPH1-Ty	RHΔ*ku80*Δ*hxgprt; TUB1:OsTIR1-3FLAG, SAG1:CAT; CPH1-AID-3HA; CPH1:CPH1-2Ty, DHFR-TS:DHFR*	complementation
37	CPH1-AID/CPH1$^{\Delta Ank1}$-Ty	RHΔ*ku80*Δ*hxgprt; TUB1:OsTIR1-3FLAG, SAG1:CAT; CPH1-AID-3HA; CPH1:CPH1*$^{\Delta(48-91)}$*-2Ty, DHFR-TS:DHFR*	Complementation
38	CPH1-AID/CPH1$^{\Delta Ank2}$-Ty	RHΔ*ku80*Δ*hxgprt; TUB1:OsTIR1-3FLAG, SAG1:CAT; CPH1-AID-3HA; CPH1:CPH1*$^{(429-463)}$*-2Ty, DHFR-TS:DHFR*	Complementation
39	CPH1-BirA	RHΔ*ku80*Δ*hxgprt*; *CPH1-BirA-3HA, DHFR-TS:HXGPRT*	Biotinylation
40	RNG2-BirA	RHΔ*ku80*Δ*hxgprt*; *RNG2-BirA-3HA, DHFR-TS:HXGPRT*	Biotinylation
41	BirA-RNG2	RHΔ*ku80*Δ*hxgprt*; *DHFR-TS:DHFR, 2Ty-BirA-RNG2*	Biotinylation
42	MyoH-BirA	RHΔ*ku80*Δ*hxgprt*; *MyoH-BirA-3HA,*	Biotinylation

		DHFR-TS:HXGPRT	
43	CPH1-6HA/CaM1-2Ty	RHΔ*ku80*Δ*hxgprt*; *CPH1-6HA*, *DHFR-TS:DHFR; CaM1-2Ty, DHFR-TS:HXGPRT*	Super-resolution, immuno-EM
44	CPH1-6HA/Ty-RNG2	RHΔ*ku80*Δ*hxgprt*; *CPH1-6HA*, *DHFR-TS:HXGPRT; DHFR-TS:DHFR, RNG2:2Ty-RNG2*	Super-resolution, Immuno-EM
45	CPH1-AID/MyoH-Ty	RHΔ*ku80*Δ*hxgprt; TUB1:TIR1-3FLAG, SAG1:CAT; CPH1-AID-3HA; MyoH-2Ty, DHFR-TS:HXGPRT*	Protein stabililty
46	CPH1-AID/CIP1-Ty	RHΔ*ku80*Δ*hxgprt; TUB1:TIR1-3FLAG, SAG1:CAT; CPH1-AID-3HA; CIP1-2Ty, DHFR-TS-HXGPRT*	Protein stability
47	CPH1-AID/CIP2-Ty	RHΔ*ku80*Δ*hxgprt; TUB1:TIR1-3FLAG, SAG1:CAT; CPH1-AID-3HA; CIP2-2Ty, DHFR-TS-HXGPRT*	Protein stability
48	CPH1-AID/CIP3-Ty	RHΔ*ku80*Δ*hxgprt; TUB1:TIR1-3FLAG, SAG1:CAT; CPH1-AID-3HA; CIP3-2Ty, DHFR-TS-HXGPRT*	Protein stability
49	CPH1-AID/Ty-RNG2	RHΔ*ku80*Δ*hxgprt; TUB1:TIR1-3FLAG, SAG1:CAT; CPH1-AID-3HA; 2Ty-RNG2, DHFR-TS-HXGPRT*	Protein stability
50	CPH1-AID/DCX-Ty	RHΔ*ku80*Δ*hxgprt; TUB1:TIR1-3FLAG, SAG1:CAT; CPH1-AID-3HA; DCX-2Ty, DHFR-TS-HXGPRT*	Protein stability
51	Δ*cip1*	RHΔ*ku80*Δ*hxgprt; TUB1:TIR1-3FLAG, SAG1:CAT;* Δ*cip1::DHFR-TS:HXGPRT*	Knockdown
52	Δ*cip2*	RHΔ*ku80*Δ*hxgprt; TUB1:TIR1-3FLAG, SAG1:CAT;* Δ*cip2::DHFR-TS:HXGPRT*	Knockdown
53	Δ*cip3*	RHΔ*ku80*Δ*hxgprt; TUB1:TIR1-3FLAG, SAG1:CAT;* Δ*cip3::DHFR-TS:DHFR*	Knockdown
54	Δ*cip1*/Δ*cip2*	RHΔ*ku80*Δ*hxgprt; TUB1:TIR1-3FLAG, SAG1:CAT;* Δ*cip1;* Δ*cip2*	Knockdown
55	Δ*cip1*/Δ*cip2*Δ*cip3*	RHΔ*ku80*Δ*hxgprt; TUB1:TIR1-3FLAG, SAG1:CAT;* Δ*cip1;* Δ*cip2;* Δ*cip3::DHFR-TS:DHFR*	Knockdown
56	CPH1-AID/270770 -Ty	RHΔ*ku80*Δ*hxgprt; TUB1:TIR1-3FLAG, SAG1:CAT; CPH1-AID-3HA; TGME49_ 270770-2Ty, DHFR-TS-HXGPRT*	IFA localization
57	CPH1-AID/306220 -Ty	RHΔ*ku80*Δ*hxgprt; TUB1:TIR1-3FLAG, SAG1:CAT; CPH1-AID-3HA; TGME49_ 306220-2Ty, DHFR-TS-HXGPRT*	IFA localization
58	CPH1-AID/230340 -Ty	RHΔ*ku80*Δ*hxgprt; TUB1:TIR1-3FLAG, SAG1:CAT; CPH1-AID-3HA; TGME49_ 230340-2Ty, DHFR-TS-HXGPRT*	IFA localization
59	CPH1-	RHΔ*ku80*Δ*hxgprt; TUB1:TIR1-3FLAG,*	IFA localization

	AID/273760 -Ty	*SAG1:CAT; CPH1-AID-3HA; TGME49_ 273760-2Ty, DHFR-TS-HXGPRT*	
60	CPH1-AID/320030 -Ty	RHΔ*ku80*Δ*hxgprt; TUB1:TIR1-3FLAG, SAG1:CAT; CPH1-AID-3HA; TGME49_ 320030-2Ty, DHFR-TS-HXGPRT*	IFA localization
61	CPH1-AID/255895 -Ty	RHΔ*ku80*Δ*hxgprt; TUB1:TIR1-3FLAG, SAG1:CAT; CPH1-AID-3HA; TGME49_ 255895-2Ty, DHFR-TS-HXGPRT*	IFA localization

[1] Gene numbers were listed in Supplementary Table 1.

Supplementary Table 3. Plasmids used in the study.

	Plasmid name[1]	Genotype	Application
1	pCas9-54 sgRNA 3’	*SAG1:Cas9-GFP[2], U6:54sgRNA 3’*	C-terminal tagging
2	pCas9-54 sgRNA 5’	*SAG1:Cas9-GFP, U6:54sgRNA 5’*	5’ targeting
3	pCas9-54 sgRNA 5’/3’	*SAG1:Cas9-GFP, U6:54sgRNA 5’, U6: 54sgRNA 3’*	Knockout
4	pCas9-35 sgRNA 3’	*SAG1:Cas9-GFP, U6:35sgRNA 3’*	C-terminal tagging
5	pCas9-35 sgRNA 5’	*SAG1:Cas9-GFP, U6:35sgRNA 5’*	5’ targeting
6	pCas9-35 sgRNA 5’/3’	*SAG1:Cas9-GFP, U6:35sgRNA 5’, U6: 35sgRNA 3’*	Knockout
7	pCas9-03 sgRNA 3’	*SAG1:Cas9-GFP, U6:03sgRNA 3’*	C-terminal tagging
8	pCas9-03 sgRNA 5’	*SAG1:Cas9-GFP, U6:03sgRNA 5’*	5’ targeting
9	pCas9-03 sgRNA 5’/3’	*SAG1:Cas9-GFP, U6:03sgRNA 5’, U6: 03sgRNA 3’*	Knockout
10	pCas9-58 sgRNA 3’	*SAG1:Cas9-GFP, U6:58sgRNA 3’*	C-terminal tagging
11	pCas9-58 sgRNA 5’	*SAG1:Cas9-GFP, U6:58sgRNA 5’*	5’ targeting
12	pCas9-58 sgRNA 5’/3’	*SAG1:Cas9-GFP, U6:58sgRNA 5’, U6: 58sgRNA 3’*	Knockout
13	pCas9-66 sgRNA 3’	*SAG1:Cas9-GFP, U6:66sgRNA 3’*	C-terminal tagging
14	pCas9-66 sgRNA 5’	*SAG1:Cas9-GFP, U6:66sgRNA 5’*	5’ targeting
15	pCas9-66 sgRNA 5’/3’	*SAG1:Cas9-GFP, U6:66sgRNA 5’, U6: 66sgRNA 3’*	Knockout
16	pCas9-51sgRNA 3’	*SAG1:Cas9-GFP, U6:51sgRNA 3’*	C-terminal tagging
17	pCas9-51 sgRNA 5’	*SAG1:Cas9-GFP, U6:51sgRNA 5’*	5’ targeting
18	pCas9-51 sgRNA 5’/3’	*SAG1:Cas9-GFP, U6:51sgRNA 5’, U6: 51sgRNA 3’*	Knockout
19	pCas9-37 sgRNA 3’	*SAG1:Cas9-GFP, U6:37sgRNA 3’*	C-terminal tagging
20	pCas9-37 sgRNA 5’	*SAG1:Cas9-GFP, U6:37sgRNA 5’*	5’ targeting
21	pCas9-37 sgRNA 5’/3’	*SAG1:Cas9-GFP, U6:37sgRNA 5’, U6: 35sgRNA 3’*	Knockout
22	pCas9-22 sgRNA 3’	*SAG1:Cas9-GFP, U6:22sgRNA 3’*	C-terminal tagging

23	pCas9-22 sgRNA 5’	*SAG1:Cas9-GFP, U6:22sgRNA 5’*	5’ targeting
24	pCas9-22 sgRNA 5’/3’	*SAG1:Cas9-GFP, U6:22sgRNA 5’, U6: 35sgRNA 3’*	Knockout
25	pCas9-34 sgRNA 3’	*SAG1:Cas9-GFP, U6:34sgRNA 3’*	C-terminal tagging
26	pCas9-34 sgRNA 5’	*SAG1:Cas9-GFP, U6:34sgRNA 5’*	5’ targeting
27	pCas9-34 sgRNA 5’/3’	*SAG1:Cas9-GFP, U6:34sgRNA 5’, U6: 35sgRNA 3’*	Knockout
28	pCas9-41 sgRNA 3’	*SAG1:Cas9-GFP, U6:41sgRNA 3’*	C-terminal tagging
29	pCas9-41 sgRNA-5’	*SAG1:Cas9-GFP, U6:41sgRNA 5’*	5’ targeting
30	pCas9-41 sgRNA 5’/3’	*SAG1:Cas9-GFP, U6:41sgRNA 5’, U6: 41sgRNA 3’*	Knockout
31	pCas9-67 sgRNA 3’	*SAG1:Cas9-GFP, U6:67sgRNA 3’*	C-terminal tagging
32	pCas9-67 sgRNA 5’	*SAG1:Cas9-GFP, U6:67sgRNA 5’*	5’ targeting
33	pCAs9-67 sgRNA 5’/3’	*SAG1:Cas9-GFP, U6:67sgRNA 5’, U6:67sgRNA 3’*	Knockout
34	pCas9-27 sgRNA 3’	*SAG1:Cas9-GFP, U6:27sgRNA 3’*	C-terminal tagging
35	pCas9-69 sgRNA 3’	*SAG1:Cas9-GFP, U6:69sgRNA 3’*	C-terminal tagging
36	pCas9-95 sgRNA 3’	*SAG1:Cas9-GFP, U6:95sgRNA 3’*	C-terminal tagging
37	pCas9-79 sgRNA 3’	*SAG1:Cas9-GFP, U6:79sgRNA 3’*	C-terminal tagging
38	pCas9-93 sgRNA 3’	*SAG1:Cas9-GFP, U6:93sgRNA 3’*	C-terminal tagging
39	pCas9-56 sgRNA 3’	*SAG1:Cas9-GFP, U6:56sgRNA 3’*	C-terminal tagging
40	pCas9-31 sgRNA 3’	*SAG1:Cas9-GFP, U6:31sgRNA 3’*	C-terminal tagging
41	pCas9-52 sgRNA 3’	*SAG1:Cas9-GFP, U6:52sgRNA 3’*	C-terminal tagging
42	pCas9-70 sgRNA 3’	*SAG1:Cas9-GFP, U6:70sgRNA 3’*	C-terminal tagging
43	pCas9-74 sgRNA 3’	*SAG1:Cas9-GFP, U6:74sgRNA 3’*	C-terminal tagging
44	pCas9-RNG2 sgRNA 3’	*SAG1:Cas9-GFP, U6:RNG2sgRNA 3’*	C-terminal tagging
45	pCas9-RNG2	*SAG1:Cas9-GFP, U6:RNG2sgRNA*	N-terminal tagging

	sgRNA 5’	*5’*	
46	pCas9-DCX sgRNA 3’	*SAG1:Cas9-GFP, U6:DCXsgRNA 3’*	C-terminal tagging
47	pCas9-CIP1 sgRNA 5’	*SAG1:Cas9-GFP, U6:CIP1sgRNA 5’*	Knockout
48	pCas9-CIP1 sgRNA 3’	*SAG1:Cas9-GFP, U6:CIP1sgRNA 3’*	C-terminal tagging
49	pCas9-CIP1 sgRNA 5’/3’	*SAG1:Cas9-GFP, U6:CIP1sgRNA 5’, U6:CIP1sgRNA 3’*	Knockout
50	pCas9-CIP2 sgRNA 5’	*SAG1:Cas9-GFP, U6:CIP2sgRNA 5’*	Knockout
51	pCas9-CIP2 sgRNA 3’	*SAG1:Cas9-GFP, U6:CIP2sgRNA 3’*	C-terminal tagging
52	pCas9-CIP2 sgRNA 5’/3’	*SAG1:Cas9-GFP, U6:CIP2gRNA 5’, U6:CIP2gRNA 3’*	Knockout
53	pCas9-CIP3 sgRNA 5’	*SAG1:Cas9-GFP, U6:CIP3sgRNA 5’*	Knockout
54	pCas9-CIP3 sgRNA 3’	*SAG1:Cas9-GFP, U6:CIP3sgRNA 3’*	C-terminal tagging
55	pCas9-CIP3 sgRNA 5’/3’	*SAG1:Cas9-GFP, U6:CIP3gRNA 5’, U6:CIP3gRNA 3’*	Knockout
56	pCas9-270770 sgRNA 3’	*SAG1:Cas9-GFP, U6:* TGME49_*270770sgRNA 3’*	C-terminal tagging
57	pCas9-306220 sgRNA 3’	*SAG1:Cas9-GFP, U6:* TGME49_306220*sgRNA 3’*	C-terminal tagging
58	pCas9-230340 sgRNA 3’	*SAG1:Cas9-GFP, U6:* TGME49_ *230340sgRNA 3’*	C-terminal tagging
59	pCas9-273760 sgRNA 3’	*SAG1:Cas9-GFP, U6:* TGME49_*273760sgRNA 3’*	C-terminal tagging
60	pCas9-320030 sgRNA 3’	*SAG1:Cas9-GFP, U6:* TGME49_*320030 sgRNA 3’*	C-terminal tagging
61	pCas9-255895 sgRNA 3’	*SAG1:Cas9-GFP, U6:* TGME49_*255895sgRNA 3’*	C-terminal tagging
62	pCas9-MyoH sgRNA 3’	*SAG1:Cas9-GFP, U6:MyoHsgRNA 3’*	C-terminal tagging
63	pN-BirA-DHFR	*DHFR:DHFR-TS, RNG2:Ty-BirA*	N-terminal tagging
64	pN-2Ty-DHFR	*DHFR:DHFR-TS, RNG2:2Ty*	N-terminal tagging
65	pDHFR-LoxP-CPH1-Ty	*DHFR:DHFR-TS, CPH1:CPH1-2Ty*	Complementation
66	pDHFR-LoxP-CPH1$^{\Delta Ank1}$-Ty	*DHFR:DHFR-TS, CPH1:CPH1*$^{\Delta(48\text{-}91)}$*-2Ty*	Complementation
67	pDHFR-LoxP-CPH1$^{\Delta Ank2}$-Ty	*DHFR:DHFR-TS, CPH1:CPH1*$^{\Delta(429\text{-}463)}$*-2Ty*	Complementation
68	pLinker-2Ty-HXGPRT-LoxP	*Linker-2Ty, LoxP-DHFR-TS:HXGPRT-LoxP*	C-terminal tagging
69	pLinker-2Ty-DHFR-LoxP	*Linker-2Ty, LoxP-DHFR-TS:DHFR-LoxP*	C-terminal tagging

70	pLinker-BirA-3HA-HXGPRT-LoxP	*Linker-BirA-3HA, LoxP-DHFR-TS:HXGPRT-LoxP*	C-terminal tagging
71	pLinker-6HA-HXGPRT-LoxP	*Linker-6HA, LoxP-DHFR-TS:HXGPRT-LoxP*	C-terminal tagging
72	pLinker-AID-3HA-DHFR-LoxP	*Linker-AID-3HA, LoxP-DHFR-TS:DHFR-TS-LoxP*	C-terminal tagging
73	pMIC2-GLuc	*TUB1:MIC2-GLuc-c-myc, DHFR-TS:HXGPRT*	Microneme secretion
74	pDsRed-DHFR	*TUB:SAG1-DsRed, DHFR:DHFR-TS*	Microneme secretion

[1] refers to gene numbers that are listed in Supplementary Table 1.
[2] SAG1 promoter driving expression of Cas9-GFP.

Supplementary Table 4. Primers used in the study.

Gene	Name[1]	Sequence (5'>………..<3')
TGGT1_254870	54sgRNA 3'	GTTGATGAAGATCAGTAAAAGTTTTAGAGCTAGAAATAGC
	54sgRNA 5'	ATACTGCGTGTGATATGCGAGTTTTAGAGCTAGAAATAGC
	54L	CTGGACTATCTATACGCCTTTTTGGAAGTTGATGAAGATCAGGCTAGCAAGGGCTCGGG
	54T	TGGCCTTGTTGTGGTTTTCGTTGAACCGTGAGCGATCCATTTATACGACTCACTATAGG
	54M	TACACTGAAAAGTGGAAAGAGCGCGATACTGCGTGTGATATGGTAAAACGACGGCCAGT
	54P	TGGATGAGGGTGAGTGAAGG
	54F	CACACGGCACCTTGAAGAC
	54R	CTCCTGCGTTCGGTGAGC
TGGT1_222350	35sgRNA 3'	GCAATAGATTTCTGTCGAGAGTTTTAGAGCTAGAAATAGC
	35sgRNA 5'	GCGCGCAAGCGGCTGCATGC GTTTTAGAGCTAGAAATAGC
	35L	GTCAAGTATCGATTTCAGGCTGCGGACCCAGAAGATCTGCAAGCTAGCAAGGGCTCGGG
	35T	CTTCGAATACATCACTTTAGGAAGAATTGGGGGAAACCTTCTATACGACTCACTATAGG
	35M	CTTCCTCCGAGTTTTCTTCTTCGGACTGCTCAAGCTCCAGCAGTAAAACGACGGCCAGT
	35P	GCGTCCGTCTGGAGAGGT
	35F	TCCACGCGACGAAGACTG
	35R	GCGGTGTACAGACACACC
TGGT1_266630	66sgRNA 3'	CTCTTCGAGACTGAAAGAAAGTTTTAGAGCTAGAAATAGC
	66sgRNA 5'	TCGCTCGTGTAGTGTTGGTGGTTTTAGAGCTAGAAATAGC
	66L	GTTTCTGAGTCTCCGGCGGCAGATTTCTGGTCTCTTCGAGACGCTAGCAAGGGCTCGGG
	66T	TGACAAGGACCATGGACAGCGCCACCTTTCAAAGTGCCTTTTATACGACTCACTATAGG
	66M	TATGTCAATCGAAAAAATTCGGTCTTCGCTCGTGTAGTGTTGGTAAAACGACGGCCAGT
	66P	CGTGTTGCCCATCCATCTGTC
	66F	GCGTGTGACTCAAGCTGTC
	66R	GCGTTCGAACGAACTGCGTC

TGGT1_320030	03sgRNA 3'	AAGTGAAAAAGCGAAGTAGGGTTTTAGAGCTAGAAATAGC
	03sgRNA 5'	ATTCGTGCTTCATTTTACTGGTTTTAGAGCTAGAAATAGC
	03L	GGTCCATTTCCAGCCGTTCCCATCAGAAGTGAAAAAGCGAAGGCTAGCAAGGGCTCGGG
	03T	TCCTGCCACGCCACGCCTATGTGGTGACCTCCTAGTCCACCTATACGACTCACTATAGG
	03M	TTCGCTCGCAGAAAAGGACTGCAATCGCTAGTTCTACCTCAGGTAAAACGACGGCCAGT
	03P	GCCATGAAGCGATCCGAG
	03F	GAACGACGCTAAGACTGG
	03R	CCGGACAGGGAGCAAGTG
TGGT1_258090	58sgRNA 3'	GTGTCTCTGTCTGCGTCTTCGTTTTAGAGCTAGAAATAGC
	58sgRNA 5'	TTGCCATCGCAACAAAAGTTGTTTTAGAGCTAGAAATAGC
	58L	CGGATCAAACAGATCCTTTTTCAGGCTCAGAGAAGAAAGAACGCTAGCAAGGGCTCGGG
	58T	TCCGTCGCTGGTGGAGAAAAGCCGTCTGTGAAACTTCCAGAAATACGACTCACTATAGG
	58M	GAGGCCGGGAGAGTGTGACAGCGGCTGACTGTACACCCGAACGTAAAACGACGGCCAGT
	58P	AGGAGGGGGAGACGTGGA
	58F	CCCGCCTTCTGTGGTTCC
	58R	TGCTGCAGCCTGCCATAC
TGGT1_315510	51g	TAAAAGCTAATGCCTCACAGGTTTTAGAGCTAGAAATAGC
	51sgRNa 5'	GAGACCGAACTCTCGCGCAGGTTTTAGAGCTAGAAATAGC
	51L	AAAATAAGAGAGGGGACCCCTCCGCCGTGGAGAGTTAAAAGCGCTAGCAAGGGCTCGGG
	51T	AAAGCGTCGAAGCACTTGATCATTTATGGAAACACACCTCTGATACGACTCACTATAGG
	51M	GCGGTTTTCACACCGGCGCTAGCGCGAGACCGAACTCTCGCGGTAAAACGACGGCCAGT
	51P	GTCATGCAGGCGAGAGTT
	51F	TGGGGCATACATGGCATGAC
	51R	GTGACAGGACACGGTTCAC
TGGT1_226990	22sgRNA 3'	CACAGAACGAGGAATTAACGGTTTTAGAGCTAGAAATAGC

	22sgRNA 5'	CGCCCACGCCATTTGAACCTGTTTTAGAGCTAGAAATAGC
	22L	ACTCTTTTAGTACCGTCGCATACGCGTATAATTGTCCCGCGTGCTAGCAAGGGCTCGGG
	22T	CGTGTGCATTCGCTCGGAACAAGGGCACAGAACGAGGAATTAATACGACTCACTATAGG
	22M	AAAAAGCAATAAAAGCAAAAACCCTGCGAGGAAGGCCAAGGGTAAAACGACGGCCAGT
	22P	CGCTGTCAGGACAGACAC
	22F	CAGCCTCACCGTCTTCAAC
	22R	ATTCTTGGTCGCCGTTGGG
TGGT1_234270	34sgRNA 3'	GTGGCGTTCTCGACAGCTCAGTTTTAGAGCTAGAAATAGC
	34sgRNA 5'	GATGCGCCATCGTGAACAACGTTTTAGAGCTAGAAATAGC
	34L	GAGGAAAAGAAAGAAGACAAGGAGTCCAAGGAAGAGAATCCGGCTAGCAAGGGCTCGGG
	34T	GCGTTTTTCGACCTCCCCTTCTGGAGTGGCGTTCTCGACAGCATACGACTCACTATAGG
	34M	TTCCCTCTCTTCGCTCACTCTTGCACCTGATTTCCCCTGTTGTAAAACGACGGCCAGT
	34P	GTCGTGTGGCGTCGAAGT
	34F	GCGTTCTGGAGTTGCTCG
	34R	TGCACCATCTGTGCGGTC
TGGT1_313780	37sgRNA 3'	GTCGCGTGCACAAATTTCATGTTTTAGAGCTAGAAATAGC
	37sgRNA 5'	TGCCGAAACGGAGGCACAGAGTTTTAGAGCTAGAAATAGC
	37L	CGACAACAAACGGGGTCGCTGTGGTGCGGATCATGTGCCCGAATTGGAAGTGGAGGACG
	37T	GCGACTGATAAACTCTTCACAGGCAGTCGCGTGCACAAATTTATACGACTCACTATAGG
	37M	TACCTCGCCTGTTGGTTTCTGAAGAGTCACACGAAGCCTTCTGTAAAACGACGGCCAGT
	37P	CAGCTGTGTACGGATCTG
	37F	GCGCTCTGTCTAGCTGTGAG
	37R	GAGTTCTACGTCCCACGCTC
TGGT1_274120	41sgRNA 3'	AGAAGCAGATGCATGTTAAGGTTTTAGAGCTAGAAATAGC
	41sgRNA 5'	GTTGCCTCCGCGCCGAAGACGTTTTAGAGCTAGAAATAGC

	41L	GCCGACTCAGGCATAACCGAACAAGCCATCAGCCGGAAACAGGCTAGCAAGGGCTCGGG
	41T	TCTGCAGACACGGCTAGATACACACAGAAGCAGATGCATGTTATACGACTCACTATAGG
	41M	TCCAAGTTACCGCCTAGGCAAGGCAGAAGCCACTTCCCCGTCGTAAAACGACGGCCAGT
	41P	GACGCCTCTCCTGTTGAT
	41F	GTGGGGAGGCAGAGAAGG
	41R	CAAGTCATGTCCCAATCAGC
TGGT1_246720	67sgRNA 3'	GGGGAAAATCTTGTTGAGTGGTTTTAGAGCTAGAAATAGC
	67L	TACCACGGCCACGGAAATATCATCACATGGGGAAAATCTTGTGCTAGCAAGGGCTCGGG
	67T	ATACGAAGCCTCTGACATCCAAACCTCTTTCGCCCTCCACAC ATACGACTCACTATAGG
	67P	CGTGGTGGACAAGCTGAC
TGGT1_248740	74sgRNA 3'	AGGCGCATAGTAGCAAACGAGTTTTAGAGCTAGAAATAGC
	74L	CTGAGTGTCCAGAATACGGCGGTCAAAGCAAACGAAGGCGCAGCTAGCAAGGGCTCGGG
	74T	ACCCTTGTTCGGTATACTCCCGTTTGCTACCATCCGCCTTCGATACGACTCACTATAGG
	74P	GAACGGACAGTGGGTTGC
TGGT1_269330	69sgRNA 3'	GACTCTTGCATTCAGTTGAG GTTTTAGAGCTAGAAATAGC
	69L	CTCCGTGCTGGTCGCAACCGGTTACACATTGAGCCTCTCAACGCTAGCAAGGGCTCGGG
	69T	TGATAGCCACAGTATGCAAGAAGCAGACTCTTGCATTCAGTTATACGACTCACTATAGG
	69P	GAAGCCAGCTCCTGTCCG
TGGT1_231070	70sgRNA 3'	TTCGTATGCTGACATCCCGA
	70L	AACCTCGCCGTTGTGTCAGCGGCATCAGCTGACTTCGTATGC
	70T	ATTGCATGTGGACTGCCATCGAAGCTCTTGCAATCTCCGTCG
	70P	CTGTCTTCTGCGGGAAAG
TGGT1_227000	27sgRNA 3'	ATCACACGTAGAAACATCGCGTTTTAGAGCTAGAAATAGC
	27L	CTGATGTCTGGGCAGACGGTCACTGAGTTGGCGGATCACACGGCTAGCAAGGGCTCGGG

	27T	TTTTCGTCTGTCTCCACGAGTACCGAGAGTGTCGATCCTG CGATACGACTCACTATAGG
	27P	GCGATACTGCAAAGGCAGC
TGGT1 _29542 0	95sgRNA 3'	TCCCCTCGCATGCAATCGTGGTTTTAGAGCTAGAAATAGC
	95L	GTGAAAGGCATAGATTTGCTAGATTCCCCTCGCATGCAAT CGGCTAGCAAGGGCTCGGG
	95T	AGAACGTGCACAAGGAACGACGCCTAAGAGACGGCACCT CACATACGACTCACTATAGG
	95P	GCTGGTTGTTGACGAAGTC
TGGT1 _22379 0	79sgRNA 3'	CGCACACACAAGTTCTAAGGGTTTTAGAGCTAGAAATAGC
	79L	CGGGACCGTCCTGTCTTCGGTCGTCAGCGCACACACAAG TTCGCTAGCAAGGGCTCGGG
	79T	GATCTGGATTCTTCCGGTGAAGAAAGGGGACGCTCTCCT CCTATACGACTCACTATAGG
	79P	CGTGTCTTACGGAGCCAG
TGGT1 _29354 0	93sgRNA 3'	GACTCTTGCATTCAGTTGAGGTTTTAGAGCTAGAAATAGC
	93L	CTCCGTGCTGGTCGCAACCGGTTACACATTGAGCCTCTCA ACGCTAGCAAGGGCTCGGG
	93T	TGATAGCCACAGTATGCAAGAAGCAGACTCTTGCATTCAG TTATACGACTCACTATAGG
	93P	GAAGCCAGCTCCTGTCCG
TGGT1 _24564 0	56sgRNA 3'	GATGCGGATCGGCTGGTAACGTTTTAGAGCTAGAAATAGC
	56L	GGTTCCGTGCTGGACATTGGCCACATGATGCGGATCGGC TGGGCTAGCAAGGGCTCGGG
	56T	TAAAGCATGGCAAAAAATCGATTAGAAAACTTTGTTCCTGT TATACGACTCACTATAGG
	56P	CTATGGTACGCTGCAGCC
TGGT1 _31215 0	31sgRNA 3'	ATAAATATTTGTTCTTTCTCGTTTTAGAGCTAGAAATAGC
	31L	GTCGTTGTCACATCAGACTGTGTCTCCGGAGCTACTAAAG TCGCTAGCAAGGGCTCGGG
	31T	TTGCCCTGGGTGTCTGCGGTCCAAAATAAATATTTGTTCTT TATACGACTCACTATAGG
	31P	GACATGCAGAGCAGCAAG
TGGT1 _30527 0	52sgRNA 3'	TTCAAGGAAAAAAGTTATTAGTTTTAGAGCTAGAAATAGC

	52L	GGCACATCGTTGAGAATCCTCCAGTCGCCGTTTGCGCAG GAGGCTAGCAAGGGCTCGGG
	52T	CGAATCGAACACTGTAATACATTTGTTCAAGGAAAAAGTT AATACGACTCACTATAGG
	52P	GCAGAAGTCGGCATCCTC
N-RNG2	sgRNA 5'	AAGGTGGGGGTGCATGGTGGGTTTTAGAGCTAGAAATAG C
	M	ATGTAAGGCCAGATTCCGAATTCTTTGGGTGACGTTCCGC CAGTAAAACGACGGCCAGT
	O	GAAGGCTCTTGAGGCTCTGCGGAAGAAAGGTGGGGGTGC ATTTTGTCCGATGCCGAGCC
	F	GCTCCTCGAACTCCGTAG
RNG2-C	sgRNA 3'	CAAACTAAAAAAAGGGATCGGTTTTAGAGCTAGAAATAGC
	L	GACGCGCCTATCCAGCGTGTTGTCTCGGACGCATCAACA AACGCTAGCAAGGGCTCGGG
	T	GAAGGCAATTTCTCTTTCGGCCGGTCATTTTAAGTTCCAC GAATACGACTCACTATAGG
	P	CGTGCAAACCGTGGAAAG
AKMT1	sgRNA 3'	CAATTGTGGGAGACTGGCGGGTTTTAGAGCTAGAAATAGC
	L	GGAACCAAAAAAACTGCATGCGAGGAACGCCTACCGGCC AGTGCTAGCAAGGGCTCGG
	T	ACACTGAGCCCTTTACCCCTTGCGAACACGAGAAGTCCTC CGATACGACTCACTATAGG
	P	GGTCACAGGTCATGCCTC
TGGT1_234250	CIP1sgRNA 3'	TCGGCGGCTCCGAAGACCTAGTTTTAGAGCTAGAAATAGC
	CIP1 L	CTGCCTGCTGCAGTGGCAAAAGTGTCGGCGGCTCCGAAG ACCGCTAGCAAGGGCTCGGG
	CIP1 T	TTCGCGGCGTCCTGGTGGTACGATCTTCTCCATGTTGCCC TAATACGACTCACTATAGG
	CIP1 P	CACCGAGGCAACCTCCTC
	CIP1sgRNA 5’	AGAAGACAGGAGGCTGAGGGTTTTAGAGCTAGAAATAGC
	CIP1 M	CAGGGAAGCACAACCTGAGGAACGGAGAAGACAGGAGG CTGA TCACTCGAGGGTCGACGG
	CIP1 H	CAGGGAAGCACAACCTGAGGAACGGAGAAGACAGGAGG CTGA CTACTCGAGGGTCGACGG
	CIP1 R	GCCTCAGGCTCACCTCTC
	CIP1 F	ACGTGAGCAACTCTCCAG
	CIP1 E	GACGTCACCGAGTCTGGC
	CIP1 S	GCTCTCTTCTCGGACTGC
TGGT1	CIP3_sgR	

_225020	NA 3'	AGGTGTGGATTCAGACGGGAGTTTTAGAGCTAGAAATAGC
	CIP3_L	CGTGGAAACCGACAACTCTGTTGACGGCTACGTTTGCGAAGCTAGCAAGGGCTCGG
	CIP3_T	CGTCCACCTGACAATTTGTCCACCACGTACAACCGCCATCCAATACGACTCACTATAGG
	CIP3_P	CACCGAGGCACACTTCCA
	CIP3_sgRNA 5'	TGAAAGCGATGTGCTGGGGAGTTTTAGAGCTAGAAATAGCAAG
	CIP3 M	GCTCTGTGTGTTCCCTCCCTTTCTTCAATCTCTTAACCGTCCGTAAAACGACGGCCAGT
	CIP3 H	GCTCTGTGTGTTCCCTCCCTTTCTTCAATCTCTTAACCGTCCCTACTCGAGGGTCGACGG
	CIP3 F	ATCTGTTCGGCCGACGTC
	CIP3 R	CTTGGCGAGTGCACTTAG
	CIP3 E	CGAGACACCGAACAACGC
	CIP3 S	TGAATGTCGGAGCATCTGG
TGGT1_257300	CIP2sgRNA 5’	CTGGGATACGGCTGTCTGGGTTTTAGAGCTAGAAATAGC
	CIP2 M	GTTTCTCCTTCAAGATGAATTGCTCCTGGGATACGGCTGTCTTCACTCGAGGGTCGACGG
	CIP2 H	
	CIP2 F	GTCCAGGGTAGTCGCTCT
	CIP2 R	ATGTCCACATCGGCTGTG
	CIP2 E	GCCTCTACTGCATTGCGT
	CIP2 S	GCCCATCACTGCCACGACG
	CIP2_sgRNA 3'	CAAGGGGTTTCGAACATGGCGTTTTAGAGCTAGAAATAGC
	CIP2_L	CGCTCAAGAAAGCGGCGCCCTCGGGACCGCCCAAGAAGGCAGCTAGCAAGGGCTCGG
	CIP2_T	ACGCAGATCCTAGAGGCTGAAAGAAGAGATACCTGCCCGCCAATACGACTCACTATAGGG
	CIP2_P	GAACGCGAAACTCGATCC
TGME49_270770	270770_S sgRNA 3’	AAGAAGAAGCGGCGAGTCAGGTTTTAGAGCTAGAAATAGCAAG
	L	CTACTCATCTACGAGCAGCTCAAGCTGAAGGAGTGCTCTGAGGCTAGCAAGGGCTCGGG
	T	GTTCCGGACTTGGCCTCACTCTCAACCTCCCTGGGTCCGCTG AATACGACTCACTATAGG
TGME49_306220	306220_sgRNA 3’	ACTAGAGGAGAGACCGCGGAGTTTTAGAGCTAGAAATAGCAAG
	L	GAAAGGGCAGGAAAGACGACGGCAAGCGAAAAAGAAGAC

		ACGCTAGCAAGGGCTCGGG
	T	TACGAAAACGTCCTTTCCTCGTCGAGGCTTCCCTTCCGCG G AATACGACTCACTATAGG
TGME49_230340	230340_sgRNA 3’	AACAGTGAAGAAGAGTGCCG GTTTTAGAGCTAGAAATAGCAAG
	L	ACCAAGAATGTCTCTGGGCAGCTCCGGAGTCTTCAGAAACAG GCTAGCAAGGGCTCGGG
	T	GGAAAAAAGTATGTCCCTAATAATCTCGAGTGTCTCCCGCGG AATACGACTCACTATAGG
TGME49_273760	273760_sgRNA 3’	CGCACATGCATCAACTTCCGGTTTTAGAGCTAGAAATAGCAAG
	L	GGCATGGGAGGCTCTGGCGGCCCCACCGTGGAGGAAGTTGATGCTAGCAAGGGCTCGGG
	T	TATATACACCAGGACTCCCCCTCTTCAACCTCTCTTCCACGG AATACGACTCACTATAGG
TGME49_320030	320030_sgRNA 3’	GTCACCACATAGGCGTGGCGGTTTTAGAGCTAGAAATAGCAAG
	L	GGTCCATTTCCAGCCGTTCCCATCAGAAGTGAAAAAGCGAAGGCTAGCAAGGGCTCGGG
	T	GCTGTTTGAAGTCACGGAAGCATGTCTGCACTCCTGCCACGC AATACGACTCACTATAGG
TGME49_255895	255895_sgRNA 3’	AAAGTTGACGACCGTGGCTCGTTTTAGAGCTAGAAATAGCAAG
	L	TCTCCCGCTGAGCCACTAGCTGACTACGCCGACGAAAAAAGTGCTAGCAAGGGCTCGGG
	T	ACTCTCTGCTGTGGCATCTTCTTTCTGTTGCCTCTTCCGGAGAATACGACTCACTATAGG
	5R	aatgtccacgtagttcgcgc
	3F	TAGGCTCCGACCACGAAG
Set 1	CPH1UTR F	GGTTCGGCGGCCGCCATCGCTGGGATTCTTCC
	CPH1UTR R	CGGGCCCCCTAGGTTTCTCCTCACCAACACTAC
Set 2	CPH1CDS F	GAGAAACCTAGGGACAAAATGAGTCGAGCATACGCGG
	CPH1CDS R	TGCTAGCGGGCCCGTCTCGAAGAGACCAGAAATC
Set 3	CPH1Ky1 F	ATGGCAGTGGAGCTAATTGAG
	CPH1Ky1 R	CATATCGTTGCACGCTTTTGC
Set 4	CPH1Ky2 F	TATACCCTCATGCAGCATCGC

	CPH1Ky2 R	GTAGAACTGACTGCACTCAC
	CPH1Seq 1	GGTCTTCGCTCGTGTAGTG
	CPH1Seq 2	CTCGCAGGGATACTTGACG
Set 5	RNG2 5UTR F	GTTCGGCGGCCGCGGGTACCGTTTTACAAC
	RNG2 5UTR R	TAGCCCTAGGGGTGGCGGAACGTCACC
Set 6	BirA-N F	AGGGCTAGCGACAAAATGGAGGTCCACACGAACCAGGACCCGCTCGATGACAAGGACAAC
	BirA-N-R	ACGGGCCCTTTGTCCGATGCCGAGCCCGATCCCTTGATATCCTTCTCTGCGCTTCT
Set 7	Ty F	ACGAACCAGGACCCGCTCGATGGATCGGGCTCGGCATC
	Ty R	GTGGACCTCCATATCGAGCGGGTCCTG
Set 8	DsRed F	AAGCTTGATATCAAGCTTCGCCAGGCTGT
	DsRed R	ACGGTGATTAATTAAAAAGCTCATAAAGTCGCG
	sgRNA R	AACTTGACATCCCCATTTACCAG
	HX-R	AACTAGTGGATCCGAGCAC
	DHFR-5'R	CGGCCGACAGGACGCTACTG

[1] numbers used in the beginning of primer names refer to those listed in Supplementary Table 1.

Supplementary Table 5. Antibodies used in the study for IFA, western blot and immunoEM localization.

Antibodies	Source	Catalog No.
Mouse monoclonal anti-Ty1 (clone BB2)	[1]	N/A
Rabbit polyclonal anti-HA	ThermoFisher Scientific	Cat#71-5500
Mouse monoclonal anti-HA (clone 16B12)	BioLegend	Cat#901501
Rat monoclonal anti-HA (clone 3F10)	Roche	Cat#11867423001
Rabbit polyclonal anti-GAP45	[2]	N/A
Mouse monoclonal anti-C-myc (9E10)	BioLegend	Cat#626801
Rabbit polyclonal anti-aldolase	[3]	N/A
Rabbit polyclonal anti-MLC1	[4]	N/A
Mouse polyclonal anti-IMC1	[5]	N/A
Streptavidin Alexa Fluor-488 conjugate	ThermoFisher Scientific	Cat#S32354
IRDye 800CW Streptavidin	LI-COR	Cat#926-32230
Goat anti-Mouse IgG (H+L) Secondary Antibody Conjugated with Alexa Fluor-488	ThermoFisher Scientific	Cat#A-11001
Goat anti-Mouse IgG (H+L) Secondary Antibody Conjugated with Alexa Fluor-594	ThermoFisher Scientific	Cat#A-11005
Goat anti-Rabbit IgG (H+L) Secondary Antibody Conjugated with Alexa Fluor-488	ThermoFisher Scientific	Cat#A-11034
Goat anti-Rabbit IgG (H+L) Secondary Antibody Conjugated with Alexa Fluor-594	ThermoFisher Scientific	Cat#A-11037
Goat anti-Rabbit IgG (H+L) Secondary Antibody Conjugated with Alexa Fluor-350	ThermoFisher Scientific	Cat#A-11046
Goat anti-Rat IgG (H+L) Secondary Antibody Conjugated with Alexa Fluor-594	ThermoFisher Scientific	Cat#A-11007
IRDye 800CW Goat anti-Mouse IgG (H+L)	LI-COR	Cat#926-32210
IRDye 800CW Goat anti-Rabbit IgG (H+L)	LI-COR	Cat#926-32211
IRDye 680CW Goat anti-Mouse IgG (H+L)	LI-COR	Cat#926-68070
IRDye 680CW Goat anti-Rabbit IgG (H+L)	LI-COR	Cat#926-68071
18 nm Colloidal gold AffiniPure goat Anti-Rabbit IgG (H+L) (EM Grade)	Jackson ImmunoResearch	Cat#111-215-144
18 nm Colloidal gold AffiniPure goat Anti-Rabbit IgG (H+L) (EM Grade)	Jackson ImmunoResearch	Cat#115-215-166

Supplementary Table 6 Differences in proteins identified using RNG2 tagged with BirA at the C-terminus vs. N-terminus. [1]

**Gene id**	**Fold difference**[2]	***P* value** [2]	**ToxoDB Annotation**	**Domain analayis**
TGME49_226990	0.06	0.0031	hypothetical	microtubule-associated protein
TGME49_245640	0.06	0.047	hypothetical	No

[1] datasets were statistically analyzed using Students t-test in Scaffold v4.
[2] C-terminus vs N-terminus
[3] *P* values ≤ 0.05 were considered as significant, uncorrected

Supplementary References

1. Bastin, P., Bagherzadeh, Z., Matthews, K.R. & Gull, K. A novel epitope tag system to study protein targeting and organelle biogenesis in *Trypanosoma brucei*. *Molec. Biochem. Parasitol.* **77**, 235-239 (1996).
2. Frenal, K. et al. Functional dissection of the apicomplexan glideosome molecular architecture. *Cell Host Microbe* **8**, 343-357 (2010).
3. Starnes, G.L., Coincon, M., Sygusch, J. & Sibley, L.D. Aldolase is essential for energy production and bridging adhesin-actin cytoskeletal interactions during parasite invasion of host cells. *Cell Host Microbe* **5**, 353-364 (2009).
4. Herm-Gotz, A. et al. *Toxoplasma gondii* myosin A and its light chain: a fast, single-headed, plus-end-directed motor. *EMBO Journal* **21**, 2149-2158 (2002).
5. Wichroski, M.J., Melton, J.A., Donahue, C.G., Tweten, R.K. & Ward, G.E. Clostridium septicum alpha-toxin is active against the parasitic protozoan *Toxoplasma gondii* and targets members of the SAG family of glycosylphosphatidylinositol-anchored surface proteins. *Infect Immun* **70**, 4353-4361 (2002).
