# Supplementary material for: A conserved ankyrin repeat-containing protein regulates conoid stability, motility and cell invasion in Toxoplasma gondii
Source: Nat Commun. 2017 Dec 21;8:2236. doi: 10.1038/s41467-017-02341-2 (PMC5740107; doi:10.1038/s41467-017-02341-2)
Supplement: Supplementary file 3 — Description of Additional Supplementary Files [file 41467_2017_2341_MOESM3_ESM.pdf]

Description of Additional Supplementary Files

File Name: Supplementary Data 1
Description: Input file for SFINX analysis

File Name: Supplementary Data 2
Description: Filtered output of the interactome analyses with additional information

File Name: Supplementary Data 3
Description: Filtered output of the protein interactome analyses with datasets from CPH-1BirA or MyoH-BirA

File Name: Supplementary Data 4
Description: Gene Ontology (GO) terms of molecular function for annotated proteins identified in the CPH1-RNG2-MyoH interactome

File Name: Supplementary Data 5
Description: GO terms of Cellular Compartment and Molecular Function for annotated proteins
